# Interplay between Oxidative Stress and Metabolism in Signalling and Disease 2016

**DOI:** 10.1155/2017/7013972

**Published:** 2017-01-22

**Authors:** Eric E. Kelley, Antonio Marcus de Andrade Paes, Hariom Yadav, Celia Quijano, Adriana Cassina, Andrés Trostchansky

**Affiliations:** ^1^Department of Physiology and Pharmacology, West Virginia University, Morgantown, WV, USA; ^2^Laboratory of Experimental Physiology, Department of Physiological Sciences, Federal University of Maranhão, São Luís, MA, Brazil; ^3^Diabetes, Endocrinology and Obesity Branch, NIDDK, NIH, Bethesda, MD, USA; ^4^Departamento de Bioquímica and Center for Free Radical and Biomedical Research, Facultad de Medicina, Universidad de la República, Montevideo, Uruguay

Being frequently present in insulin-sensitive tissues and the vasculature as well as critically linked to the inflammatory response, oxidative stress is a common component of metabolic dysfunction. However, despite much effort, it remains unclear to what extent oxidative stress contributes to metabolic abnormalities such as insulin resistance, steatosis/dyslipidemia, diabetes, and allied cardiovascular disease. While the identities of the specific oxidants or reactive metabolites, the sources thereof, and the associated redox-sensitive pathways that mediate the effects of oxidative stress remain elusive, progress has been made. A portion of this progress is represented in this special issue and reveals the identification of a specific source of ROS in the synaptosome and the contributory impact of thioredoxin-interacting protein (TXNIP) on inflammasome activation. These studies are complemented by comprehensive reviews of the role of mitochondria in the neuroinflammatory response and the potential participation of protein disulfide isomerase (PDI) in metabolic syndrome-induced platelet hyperactivation. Specific contributions to this special issue are summarized as follows.

E. A. Abdel-Rahman and colleagues describe the interplay between ROS-generating systems at the synapse. In freshly isolated synaptosomes, they differentiate mitochondrial-dependent consumption of molecular oxygen from that consumed through NADPH oxidase (NOX) while concomitantly defining H_2_O_2_ generation. While alteration of cellular redox homeostasis is implicated in pathology as well as aging, this process may be dependent upon the ratio of complete to incomplete oxygen reduction by the mitochondria in context of NADPH oxidase (NOX) enzymatic activity. Based on high-resolution respirometry coupled to fluorescence or electrochemical detection, the authors demonstrated that NOX and not the mitochondria is the dominant source of synaptic H_2_O_2_. As such, this study begins to more clearly elucidate the redox-related players that contribute to a variety of physiological and pathological processes in neurons.

L. Li and C. M. Stary also focused their work on mitochondria from microglia cells in the context of cerebral ischemia. Noting that microRNAs (miRs) function to regulate mitochondrial processes in astrocytes under oxidative stress, the active role for mitochondria in microglial activation, and regulation of the microglial neuroinflammatory response by miRs, the authors postulate that miR-targeted therapies represent a viable strategy for optimizing mitochondrial function and thus improve clinical outcome following cerebral ischemia. This is supported by extensive evidence of the participation of mitochondria in the glial response to injury as well as the contributory roles for miRs in regulating the glial mitochondrial response to cerebral ischemia.

H. Lee and T. Park endeavored to push beyond the associative relationship between elevated oxidant stress and metabolic syndrome by quantifying oxidative balance score (OBS) and subsequently assessing the resultant impact in the context of genetic variation. Their study of over 6000 participants found that the highest OBS scores correlated with the lowest incidence of metabolic syndrome. Furthermore, GWAS-based pathway analysis demonstrated that the VEGF signalling pathway, glutathione metabolism, and the Rac1 pathway were contributory and thus potentially involved in the interplay between oxidant stress, genetic variation, and metabolic syndrome.

The other contributions to this special issue focused on chronic inflammation (obesity/diabetes) and the role of thiol-dependent enzymes. For example, Y. D. Xiao and colleagues describe the participation of thioredoxin-interacting protein (TXNIP) on inflammasome activation in diabetes in the context of allied ischemic acute kidney injury (AKI). The ROS sensor and endogenous inhibitor of the antioxidant thioredoxin, TXNIP, is assumed to be involved in pathogenesis of diabetes. Using a rat model of diabetes, the authors report higher expression of TXNIP in addition to an increased susceptibility to ischemia/reperfusion (I/R) injury, a process attenuated by Resveratrol. Moreover, diabetic rats subjected to renal I/R injury presented lower binding of TXNIP to NLRP3, decreasing inflammasome activation. The proposed mechanism of action, validated in cell culture experiments, is TXNIP-mediated inflammasome activation through oxidative stress and this process represents a seminal signalling mechanism driving susceptibility to AKI in diabetes.

R. S. Gaspar and collaborators comprehensively reviewed the worldwide epidemic metabolic syndrome and the contributory role for protein disulfide isomerase (PDI). Elevated rates of reactive species production are tied to enhancement of platelet hyperactivation and ischemic events in obese/metabolic patients. While PDI and peroxide tone at the platelet cell membrane have been demonstrated requisite for platelet aggregation, the relationship between PDI and platelet hyperactivation in the context of metabolic syndrome is unclear. Thus, the authors focus on the activity of PDI in metabolic syndrome in order to develop a more clear understanding of the potential participation of PDI in metabolic syndrome-induced platelet hyperactivation.

In the aggregate, this special issue enhances our understanding of the intricate interplay between metabolism and oxidative stress in metabolic diseases with particular focus on mitochondria- and inflammation-derived factors that may represent potential therapeutic targets for addressing crucial health issues.

